# Melatonin—A Powerful Antioxidant in Neurodegenerative Diseases

**DOI:** 10.3390/antiox14070819

**Published:** 2025-07-03

**Authors:** Renata Kołodziejska, Alina Woźniak, Rafał Bilski, Roland Wesołowski, Daria Kupczyk, Marta Porzych, Weronika Wróblewska, Hanna Pawluk

**Affiliations:** 1 Department of Medical Biology and Biochemistry, Faculty of Medicine, Collegium Medicum in Bydgoszcz, Nicolaus Copernicus University in Toruń, Karłowicza 24, 85-092 Bydgoszcz, Poland; rafal.bilski@cm.umk.pl (R.B.); roland@cm.umk.pl (R.W.); dariak@cm.umk.pl (D.K.); hannapawluk1@wp.pl (H.P.); 2 Student Scientific Club of Biochemistry and Bioorganic Chemistry, Department of Medical Biology and Biochemistry, Faculty of Medicine, Collegium Medicum in Bydgoszcz, Nicolaus Copernicus University in Toruń, Karłowicza 24, 85-092 Bydgoszcz, Poland; porzychmarta@gmail.com (M.P.); 316714@stud.umk.pl (W.W.)

**Keywords:** melatonin, antioxidant, neurodegenerative diseases

## Abstract

Melatonin (MEL)is an endogenous hormone with antioxidant potential that plays an important role in maintaining redox homeostasis. MEL and its derivatives directly scavenge free oxygen and nitrogen radicals. Melatonin inhibits lipid peroxidation, stimulates antioxidant enzymes, and reduces metal toxicity. It stabilizes mitochondrial activity and suppresses inflammatory signaling. It takes part in neurogenesis, neuroprotection, and modulation of the cardiovascular system. It prevents many diseases of free radical etiology, i.e., neurodegenerative and circulatory system diseases and ischemic stroke. Supplementation with this antioxidant can slow down the aging process and provide protection against diseases of the central nervous system and support the body’s natural antioxidant system. This study uses current reports from the literature and meta-analyses of the antioxidant mechanisms of melatonin and its importance in neurodegenerative diseases.

## 1. Introduction

Neurodegenerative diseases represent a significant medical challenge. They involve impaired motor or cognitive function resulting from dysfunctions in the central and peripheral nervous systems. The etiology of these diseases, such as Alzheimer’s disease (AD), Parkinson’s disease (PD), and Huntington’s disease (HD), has been closely linked to mitochondrial dysfunction. Abnormalities in mitochondrial function, such as defects in the electron transport chain (ETC), oxidative phosphorylation (OXPHOS), and disturbances in ATP production and calcium levels, are considered major factors involved in the pathogenesis of these diseases [1,2,3].

Oxidative and nitrosative stress, alongside increased neuronal apoptosis, are frequently observed in affected brain regions. Reactive oxygen species (ROS), generated during mitochondrial respiration, can interact with proteins, lipids, and mitochondrial DNA (mtDNA), impairing bioenergetic function. Such mitochondrial dysfunction is not only implicated in neurodegenerative conditions but also in age-related cardiovascular disease and ischemia–reperfusion injury [3,4].

Evidence suggests that oxidative damage induced by free radicals is widespread in the brain, especially in elderly individuals, and may contribute significantly to neurodegenerative disease development [5].

In recent years, melatonin (N-acetyl-5-methoxytryptamine, MEL), an indoleamine, has garnered attention for its potential role in maintaining mitochondrial homeostasis. This methoxyindole is primarily released by the pineal gland into the bloodstream, where it is partially bound to albumin. Melatonin can also be synthesized non-enzymatically in various peripheral tissues, including lymphocytes, skin, and the gastrointestinal tract. It is also produced in the retina, eye lens, and bone marrow, where it likely exerts local autocrine and paracrine actions [2].

Melatonin is known for regulating circadian rhythms and biological clocks. Its plasma concentration is relatively low during daylight hours (10–20 pg/mL), increasing at night and peaking between midnight and 3 AM (80–150 pg/mL) [6].

The nighttime synthesis and release of melatonin by the pineal gland are strictly controlled by the circadian clock of the suprachiasmatic nucleus (SCN) [7]. In light conditions, the SCN releases gamma-aminobutyric acid (GABA), which inhibits the paraventricular nucleus and blocks melatonin synthesis and secretion. In darkness, the SCN’s inhibitory effect is lifted. This triggers norepinephrine release from the superior cervical ganglion (SCG), which stimulates melatonin production and secretion [8].

**Scheme 1:**
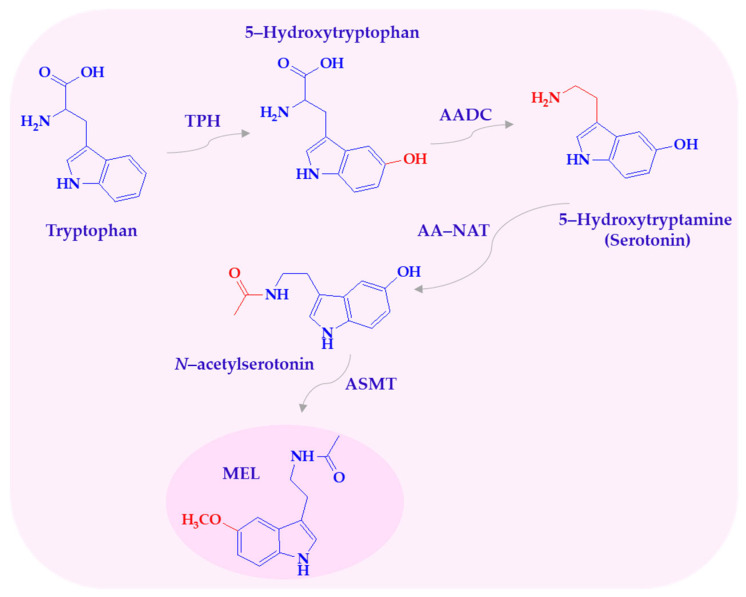
Biosynthesis of melatonin. AADC—L-aromatic amino acid decarboxylase; AA-NAT—arylalkylamine N-acetyltransferase; ASMT—N-acetylserotonin-O-methyltransferase; MEL—melatonin; TPH—tryptophan-5-hydroxylase.

Melatonin biosynthesis follows a four-step pathway involving hydroxylation, decarboxylation, acetylation, and methylation. The amino acid tryptophan is converted to serotonin, which is then acetylated to N-acetylserotonin and subsequently methylated to form melatonin (Scheme 1) [9].

Melatonin acts not only as a free radical scavenger but also reduces nitric oxide (NO) production in mitochondria, maintains electron flow, and enhances oxidative phosphorylation efficiency. It also regulates the activity of the respiratory complex, calcium influx, and intracellular secondary neurotransmitters [4], as well as mitochondrial permeability transition (mPT) and the opening of mitochondrial transition pores [1,2,3,9]. Melatonin’s effects are mediated via membrane-bound MT1 and MT2 receptors, both of which are G-protein-coupled and expressed in multiple tissues. MT1 is associated with phospholipase C (PLC) and adenylate cyclase (AC) signaling, while MT2 is involved in modulating cyclic nucleotide levels via inhibition of adenylate and guanylate cyclases [2]. Dysregulation of these receptor pathways has been implicated in various neuropsychiatric and neurodegenerative conditions, including sleep disturbances, anxiety, and depression [4].

This review explores the molecular mechanisms by which melatonin may exert neuroprotective effects, particularly in the context of mitochondrial dysfunction. Special emphasis is placed on pathways involved in oxidative stress mitigation and neuronal preservation. Understanding these mechanisms may inform future strategies for melatonin-based supplementation. Such approaches could help alleviate cognitive, motor, and sleep-related symptoms in individuals with neurodegenerative disorders. While clinical applicability remains to be fully established, the mechanistic insights presented here support the hypothesis that melatonin holds promise as a potential adjunctive agent in age-related neurological disease management.

## 2. Melatonin and Its Metabolites—Free Radical Scavengers

Melatonin contributes to antioxidant defense by attenuating oxidative stress, a condition marked by an imbalance between the production of reactive oxygen and nitrogen species (ROS/RNS) and the capacity of endogenous antioxidants to neutralize them. Excessive ROS/RNS lead to lipid peroxidation, protein oxidation, and DNA damage. These effects contribute directly to various chronic conditions, including diabetes, neurodegenerative, and cardiovascular diseases [10].

A key mechanism underlying melatonin’s antioxidant potential is its direct interaction with free radicals. Owing to its electron-rich indole ring and amphiphilic properties, melatonin crosses cellular membranes efficiently and is readily distributed within cells, enhancing its antioxidant effectiveness [11].

Furthermore, the antioxidant mechanisms of MEL differ from those of classical antioxidants, such as vitamins C and E and glutathione, which undergo the redox cycle. MEL interacts with ROS/RNS through an additive reaction, forming stable end products that are excreted in the urine. Melatonin is therefore considered a so-called ’suicidal’ or ’terminal’ antioxidant. Moreover, melatonin can interact with multiple radicals per molecule, in some cases up to ten, making it more efficient than traditional antioxidants, which typically exhibit a one-to-one stoichiometry [12,13].

**Scheme 2:**
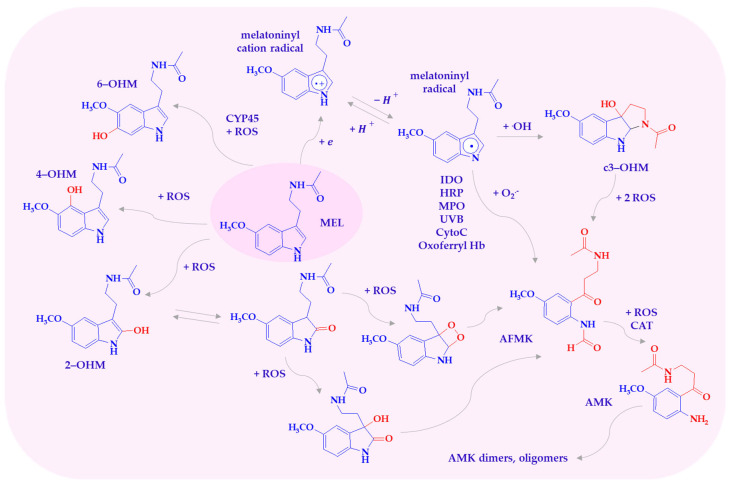
Melatonin metabolites produced by interaction with ROS. AFMK—1N-acetyl-2N-formyl-5-methoxy-nuramine; AMK—N-acetyl-5-methoxy-nuramine; c3-OHM—cyclic 3-hydroxymelatonin; CytoC—cytochrome c; HRP—hemoperoxidases; IDO—indoleamine 2,3-dioxygenase; MEL—melatonin; MPO—myeloperoxidase; 2-OHM—2-hydroxymelatonin; 4-OHM—4-hydroxymelatonin; 6-OHM—6-hydroxymelatonin; Oxoferryl Hb—oxoferryl hemoglobin.

Under high oxidative stress, melatonin is metabolized into hydroxylated derivatives and other compounds such as 1N-acetyl-2N-formyl-5-methoxykynuramine (AFMK), 1N-acetyl-5-methoxykynuramine (AMK), and cyclic 3-hydroxymelatonin (c3-OHM), either via enzymatic pathways or direct interaction with ROS/RNS (Scheme 2) [11,14]. These metabolites participate in a so-called free radical scavenging cascade, wherein they act either sequentially or in concert, and may synergize with other antioxidants like vitamins C, E, and glutathione [15,16,17]. Melatonin and its metabolites neutralize a broad spectrum of reactive species. These include superoxide anions (O_2_^•−^), hydroxyl radicals (^•^OH), singlet oxygen (^1^O_2_), hydrogen peroxide (H_2_O_2_), hypochlorous acid (HOCl), peroxyl radicals (ROO^•^), nitric oxide (NO^•^), and peroxynitrite (ONOO^−^). Additionally, they exhibit reactivity toward the cationic radical 2,2′-azino-bis(3-ethylbenzthiazoline-6-sulfonic acid) (ABTS) [15,18].

Melatonin, in reaction with one of the most toxic known reactive oxygen radicals, ^•^OH, acts as a reducing agent, donating an electron to form the melatoninyl cation radical. The melatoninyl cation radical then loses a proton, transforming into the thermodynamically more stable neutral indolyl radical, which can react through many other pathways, ultimately forming AFMK [19,20]. The products of MEL oxidation by the ^•^OH radical are hydroxylated derivatives, with the OH group at positions C2, C3, C6, and C7. These derivatives form when a melatonin molecule interacts with two hydroxyl radicals. One radical abstracts a hydrogen atom, while the other attaches to MEL [21,22].

The main enzymatic product, 6-hydroxymelatonin (6-OHM), is generated in the liver by cytochrome P450 enzymes (CYP1A1, CYP1A2, CYP2C19) and is further converted to 6-hydroxymelatonin sulfate for urinary excretion [21]. Furthermore, 6-OHM also arises through non-enzymatic routes, including interactions with ^•^OH and ONOO^−^, and during melatonin photodegradation [21]. This metabolite retains antioxidant properties, including singlet oxygen and superoxide scavenging and inhibition of iron-induced lipid peroxidation [23,24,25]. Moreover, 6-OHM has been found to be a more effective scavenger of peroxyl radicals than melatonin, Trolox, caffeine, and genistein [24].

Further, 2-Hydroxymelatonin (2-OHM) or its keto tautomer, melatonin 2-indolinone, is formed through interactions with the hydroxyl radical and oxoferryl hemoglobin, as well as by the reaction of MEL with hypochlorous acid. In the presence of HOCl, the first step involves the substitution of chlorine at the C3 position and the addition of water at the C2 position, resulting in the protonated 2-hydroxy-3-chloro intermediate. Subsequent deprotonation and dehydrohalogenation lead to the formation of 2-hydroxyindole and/or its tautomer, 3-substituted-2-oxindole [11,14].

In contrast, 4-hydroxymelatonin (4-OHM), also generated via hydroxyl radical attack or UV exposure, exhibits greater antioxidant activity than 2-OHM. This is attributed to its phenolic structure, which facilitates peroxyl radical scavenging more effectively than parent melatonin [26].

Cyclic 3-hydroxymelatonin (c3-OHM) is a stable product of melatonin hydroxylation at the 3-position. The mechanism of MEL’s transformation to c3-OHM in reaction with ^•^OH involves four steps: radical addition at the 3-position of the indole ring, tautomerization of ketoamine to enol-imine, cyclization, and finally, addition of a second OH group [11]. c3-OHM can also be formed due to the oxidation of MEL by oxoferryl hemoglobin, which, in the reaction with MEL, is transformed into the corresponding reduced form [23].

Cyclic 3-hydroxymelatonin is not only a metabolite of MEL but also a very effective scavenger of hydroxyl or peroxyl radical [18,26]. The reactivity of c3-OHM towards the ^•^OH radical is higher than that of MEL, AMK, AFMK, and vitamin C. It has also been found that c3-OHM reacts with the hydroperoxyl radical ^•^OOH several orders of magnitude faster than melatonin and its metabolites [27].

It is known that 1N-acetyl-2N-formyl-5-methoxycuramine can be formed in both enzymatic and non-enzymatic metabolic pathways [28,29]. AFMK is formed through the cleavage of melatonin at the pyrrole ring. Enzymes that convert MEL to AFMK include indoleamine 2,3-dioxygenase (IDO), myeloperoxidase (MPO), hemoperoxidases (HRP), and cytochrome c [22]. AFMK is a product of MEL oxidation in reactions with oxoferryl hemoglobin, singlet oxygen, superoxide anion, and hydrogen peroxide. Oxidative cleavage of the indole ring by H_2_O_2_ also leads to AFMK, and according to Tan and others, the intermediate product may be dioxetane or epoxide [11,21,23]. AFMK can also be derived from the intermediate metabolite of MEL and cyclic 3-hydroxymelatonin, as well as through cytochrome c-catalyzed oxidation of 2-hydroxymelatonin via 2,3-dihydroxymelatonin [18,21,30].

AFMK is further metabolized into AMK, a process facilitated by peroxidase enzymes, photochemical reactions, or ROS-mediated pathways [31,32]. AMK can undergo secondary transformations, forming dimers, oligomers, or adducts with aromatic amino acids such as tyrosine and tryptophan, thus potentially modifying proteins [31,33].

Both AFMK and AMK are major melatonin metabolites in the brain and have been shown in preclinical models to reduce neuronal damage induced by hydrogen peroxide, glutamate, or amyloid-β peptide [23,29]. AFMK protects DNA and lipids from hydroxyl radical damage. Its effectiveness in protecting DNA is approximately one-fifth of that of melatonin [34]. AMFK’s resistance to other oxidants results from its lower reactivity towards free radicals, which is related to the preference for donating two electrons per molecule [31,34]. AMK, unlike AFMK, is a potent scavenger of reactive oxygen and nitrogen species. It demonstrates greater reactivity towards the carbonate radical compared to its parent indoleamine. It also acts more effectively against singlet oxygen and scavenges peroxyl radicals generated from 2,2′-azo-bis-(amidinopropane) and cationic ABTS radicals as well as neutralizing peroxyl radicals [35].

**Scheme 3:**
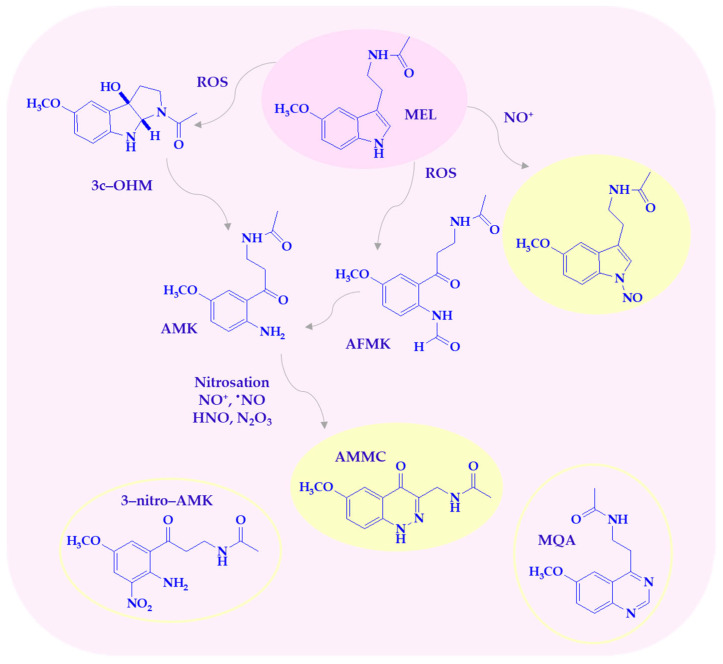
Melatonin metabolites are produced by interaction with RNS/ROS. AFMK—1N-acetyl-2N-formyl-5-methoxy-nuramine; AMK—1N-acetyl-5-methoxy-nuramine; AMMC—3-acetamidomethyl-6-methoxycinnolinone; c3-OHM—cyclic 3-hydroxymelatonin; MEL—melatonin; MQA—N-[2-(6-methoxyquinazolin-4-yl)-ethyl]-acetamide; 3-nitro-AMK—3-nitro-N-acetyl-5-methoxy-nuramine.

Melatonin and its metabolites are highly effective at scavenging reactive nitrogen species (Scheme 3), as previously discussed. Nitro-oxidative stress is caused by the overproduction of NO, which reacts with superoxide anion radical to form more reactive species, such as ONOO^−^. The peroxynitrite anion is highly toxic, contributing to lipid peroxidation, protein oxidation, DNA damage, and the induction of several transcription factors, leading to cytokine-induced chronic inflammation. MEL, as a multifunctional indoleamine, plays a crucial role in counteracting nitro-oxidative stress and shows beneficial effects against peroxynitrite-induced cellular toxicity [36]. Melatonin scavenges nitric oxide (II) through a nitrosation reaction, which is a reaction of the nitrosonium ion (NO^+^) with a nucleophile. Nitrosation and oxidation of the pyrrole nitrogen mainly lead to formation of 1-nitrosomelatonin and 1-hydroxymelatonin [37,38]. MEL eliminates the decomposition products of ONOO^−^, including OH^•,^ NO_2_^•^, and the carbonate radical (CO_3_^•−^) in the presence of physiological carbon dioxide concentrations [36]. Nitro derivatives 2-hydroxy-3-nitro- and/or 2-hydroxy-3-peroxynitro-2,3-dihydromelatonin as intermediate products can be formed in the reaction with ONOO^−^. These compounds decompose to 1,2,3,3a,8,8a-hexahydro-1-acetyl-5-methoxy-8a-hydroxypyrrolo[2,3-b]indole [11,14,36].

Among the MEL metabolites, AMK is particularly active against reactive nitrogen species. It is a powerful scavenger of all NO congeners, NO^+^, ^•^NO, and HNO, the protonated NO-subform, present at physiological pH [31,39]. One of the main AMK nitrosation products, 3-acetamidomethyl-6-methoxycinnolinone (AMMC), is more stable than 1-nitrosomelatonin due to resonance stabilization [31]. It has been demonstrated that the biological metabolites of AMK in the reaction with RNS can also be 3-nitro-AMK (AMNK, 1N-acetyl-5-methoxy-3-nitrokynuramine) and N-[2-(6-methoxyquinazolin-4-yl)-ethyl]-acetamide (MQA). Nitration of AMK to 3-nitro-AMK was observed with a mixture of peroxynitrite and bicarbonate. Peroxynitrite-CO_2_ (ONOOCO^2−)^ decomposes to a carbonate radical, CO_3_^•−^, and ^•^NO_2_, allowing the formation of 3-nitro-AMK [39]. Furthermore, AMK may suppress cyclooxygenase activity and downregulate neuronal and inducible nitric oxide synthases (nNOS, iNOS), suggesting possible anti-inflammatory properties that remain validated in clinical settings [39].

## 3. Melatonin and Its Metabolites—Regulators of Protein Expression or Activity

Melatonin exerts systemic effects due to its amphiphilic nature, allowing it to cross cellular membranes, including the blood–brain barrier (BBB) [29]. Its antioxidant and anti-inflammatory properties are particularly relevant in the context of conditions associated with oxidative stress. Melatonin functions both as a direct free radical scavenger and as an indirect modulator of oxidative defense through regulation of gene expression [12].

By interacting with various reactive oxygen and nitrogen species (ROS/RNS), melatonin may help protect cellular components, such as lipids, proteins, and DNA, from oxidative damage [14,40,41,42]. This, in turn, may contribute to reducing cellular dysfunction associated with chronic disorders [42]. In addition to its capacity to scavenge radicals, melatonin increases the production of important antioxidant enzymes in a variety of tissues, such as glutathione peroxidase (GPx), catalase (CAT), and superoxide dismutase (SOD) [43,44,45]. These enzymes work in tandem to reduce oxidative damage. SOD converts superoxide radicals into hydrogen peroxide. CAT breaks hydrogen peroxide down into water and oxygen, while GPx uses glutathione to reduce hydrogen peroxide and lipid hydroperoxides [44,45].

Melatonin influences redox homeostasis partly through activation of MT1 and MT2 receptors. MT1 receptor signaling inhibits adenylate cyclase activity, reducing cyclic adenosine monophosphate (cAMP) levels and affecting downstream protein kinase A (PKA) activity and cAMP response element-binding protein (CREB). This pathway leads to the increased expression of neurotrophic and antioxidant factors, such as brain-derived neurotrophic factor (BDNF) and nuclear factor erythroid 2 2-related factor 2 (Nrf2) [14,41,46,47]. Upon activation, Nrf2 translocates to the nucleus and binds to antioxidant response elements (AREs) in promoter regions, inducing transcription of antioxidant genes [41].

Melatonin also modulates additional transcription factors involved in oxidative and inflammatory responses, including nuclear factor kappa-light-chain-enhancer of activated B cells (NF-κB) and activator protein-1 (AP-1). Suppression of NF-κB activation has been associated with reduced inflammatory signaling, which may be beneficial in conditions where chronic inflammation contributes to tissue injury [48].

**Scheme 4:**
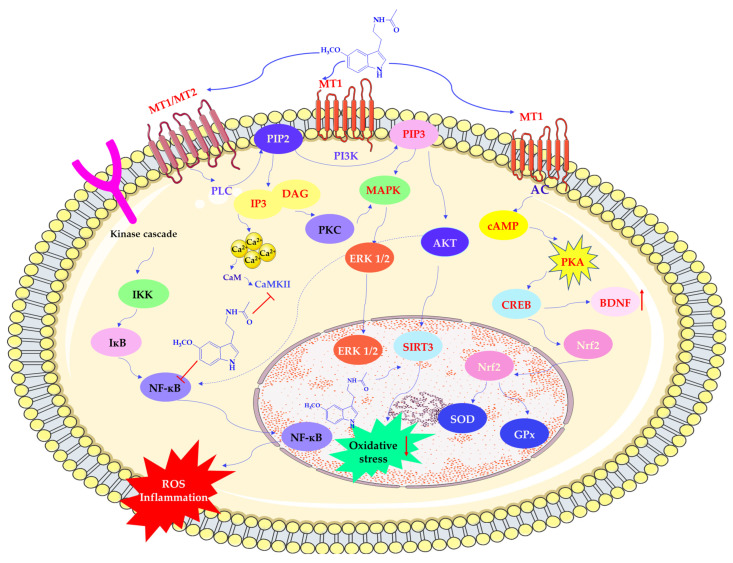
Regulation of protein expression by melatonin. AC—adenylate cyclase; AKT—protein kinase B (PKB); BDNF—brain-derived neurotrophic factor; cAMP—cyclic adenosine monophosphate; CaM—calmodulin; CaMKII—calcium/calmodulin-dependent protein kinase II; CREB—cAMP response element-binding protein; DAG—Diacylglycerol; ERK 1/2—extracellular signal-regulated kinases; GPx—glutathione peroxidase; IKK—IκB kinase; IκB—inhibitor of nuclear factor kappa B; IP3—inositol trisphosphate; MAPK—mitogen-activated protein kinase; MT1/MT2—melatonin receptor type 1 and type 2; NF-κB—nuclear factor-kappa B; Nrf2—nuclear factor erythroid 2-related factor 2; PI3K –phosphoinositide 3-kinase; PIP2—phosphatidylinositol 4,5-bisphosphate; PIP3—phosphatidylinositol 3,4,5-trisphosphate; PKA—protein kinase A; PKC—protein kinase C; PLC—phospholipase C; SOD—superoxide dismutase; SIRT3—Sirtuin 3 (silent mating type information regulation 2 homolog 3). This scheme was created using Servier Medical Art (available at https://smart.servier.com/) (accessed on 1 March 2025).

Melatonin activates PLC, which cleaves phosphatidylinositol 4,5-bisphosphate (PIP2) into diacylglycerol (DAG) and inositol triphosphate (IP3). These molecules increase intracellular calcium (Ca^2+^) concentrations. Furthermore, by binding calmodulin (CaM), MEL regulates nitric oxide synthesis. It also activates phosphoinositide 3-kinase (PI3K), which in turn stimulates protein kinase B (AKT) and mitogen-activated protein kinase (MAPK) pathways, including upregulation of Sirtuin 3 (silent mating type information regulation 2 homolog 3; SIRT3), a factor linked to mitochondrial metabolism and redox regulation [49]. Activating these signaling cascades may contribute to increased glycolysis, decreased oxidative phosphorylation, and improved cell survival under stress conditions. Additionally, melatonin influences protein kinase C (PKC), further promoting antioxidant enzyme expression and reducing the activity of pro-oxidant systems (see Scheme 4) [10,50].

An intriguing and critical extension of melatonin’s antioxidant activity is attributed to its metabolites, particularly AFMK and AMK. These metabolites are produced through enzymatic, pseudo-enzymatic, and non-enzymatic reactions of melatonin with ROS and RNS [12,18,29,44,51,52,53]. AFMK is known to scavenge aggressive radicals like hydroxyl radicals and peroxynitrite and also plays a role in preserving mitochondrial membrane potential, thereby safeguarding energy production and apoptosis regulation. AFMK has also been shown to offer neuroprotection against beta-amyloid-induced toxicity, suggesting its relevance in Alzheimer’s disease models [53].

The antioxidant capacity of AMK, the downstream metabolite of AFMK, is significantly higher. AMK’s remarkable effectiveness in neutralizing oxidative agents is reflected by its diffusion-limited reaction rates with free radicals [30,52,54,55,56]. Both AFMK and AMK have been linked to modifying inflammatory pathways, namely by inhibiting NF-κB, which lowers the generation of pro-inflammatory cytokines, in addition to radical detoxification [56,57].

By converting early ROS/RNS interactions into secondary active metabolites, the so-called “scavenger cascade”—a distinctive aspect of melatonin’s action—ensures sustained antioxidant defense. Melatonin is positioned as a complete regulator of oxidative stress due to the convergence of these many pathways, which include transcriptional modulation of antioxidant enzymes, direct scavenging, and the antioxidant activity of downstream metabolites. Its biological effects have been confirmed in organ-specific studies, not just in isolated cellular systems [5].

Melatonin treatment, for instance, decreased infarct size, enhanced neurological outcomes, and altered oxidative biomarkers in models of cerebral ischemia. Models of renal oxidative damage and cardiac ischemia–reperfusion injury have shown comparable outcomes. These organ-protective properties further support its therapeutic range. This wide range of action demonstrates the therapeutic potential of melatonin, especially for diseases where oxidative stress is a key pathogenic factor. These include aging-related physiological decline, cardiovascular illnesses (such as hypertension and ischemia–reperfusion damage), and neurodegenerative diseases (such as Alzheimer’s and Parkinson’s disease) [1,10].

## 4. Melatonin and Its Metabolites—Improvement of Mitochondrial Function

Mitochondria are essential organelles for brain cells, supporting key functions such as ion transport and neurotransmission [58]. Beyond energy production, they help regulate cell survival and apoptosis, underscoring their central role in neuronal homeostasis. Mitochondrial dysfunction is increasingly recognized as a central pathogenic factor in neurodegenerative diseases such as AD, PD, and HD. Common features of this dysfunction include impaired oxidative phosphorylation, increased ROS production, mitochondrial DNA damage, and altered organelle dynamics. Similar mitochondrial abnormalities may also be involved in systemic disorders, including cardiovascular disease, diabetes, liver conditions, and sepsis [2,58,59]. Given the brain’s high oxygen consumption and its vulnerability to redox imbalance, therapeutic strategies targeting mitochondrial integrity and ROS homeostasis are of increasing interest. As previously described, MEL is a neurohormone with antioxidant properties. In this section, we focus on its specific mitochondrial actions. These protective actions, observed in in vitro and in vivo neurodegeneration models, suggest that melatonin may act as a mitochondrial-targeted agent in brain pathology [59]. MEL is thought to support mitochondrial function through multiple complementary mechanisms beyond its well-known antioxidant capacity. Notably, MEL is taken up by mitochondria via human proton-coupled oligopeptide transporters (PEPT1/2), enabling direct access to its targets within the organelle [1]. Once inside, MEL has been reported to enhance the activity of electron transport chain complexes I, III, and IV, thereby supporting oxidative phosphorylation and ATP production [60]. Besides being imported via PEPT1/2 transporters, MEL may also be synthesized within mitochondria, as suggested by the localization of AA-NAT enzymes in the mitochondrial matrix and intermembrane space. Mitochondrial MEL synthesis appears to be favored due to the high availability of acetyl-CoA in this compartment, which exceeds the Km of AA-NAT and may promote efficient conversion of serotonin into MEL [61]. This local biosynthetic capacity may reflect an evolutionary legacy, as mitochondria are thought to have originated from melatonin-producing bacteria. These ancestral features have been retained, allowing mitochondria to maintain high melatonin concentrations independently of pineal synthesis [62]. Additionally, MEL may help to preserve mitochondrial membrane potential (Δψm), inhibit the opening of mitochondrial permeability transition pores (mPTPs), and minimize electron escape that can result in excessive ROS production [1,60]. These actions are thought to support mitochondrial efficiency and increase ATP production. By optimizing electron transport chain performance and reducing electron leakage, melatonin may help to limit free radical formation, thereby enhancing protein synthesis and cellular bioenergetics [3]. This maintenance of Δψm and mPTP closure prevents mitochondrial swelling, calcium overload, and cytochrome c release, all of which are crucial to delaying or inhibiting apoptosis, particularly in neurodegenerative conditions [60].

**Scheme 5:**
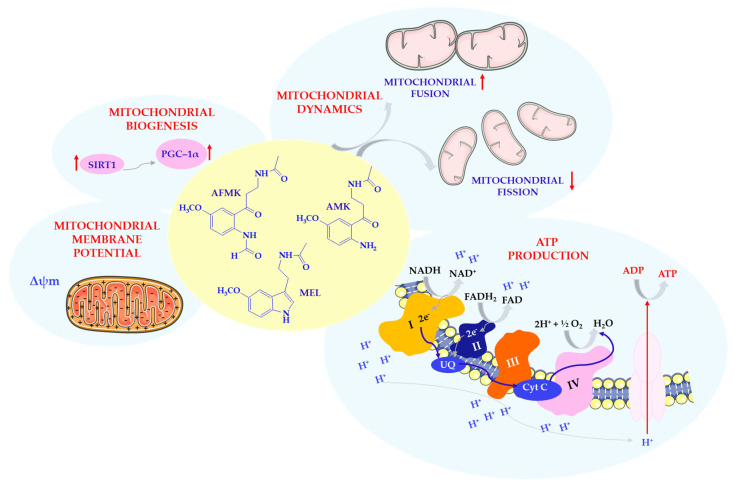
Effects of MEL and its metabolites on mitochondria. Complex I—nicotinamide adenine dinucleotide (NADH) reductase; Complex II—succinate dehydrogenase; Complex III—cytochrome reductase; Complex IV—cytochrome oxidase; Cyt C—cytochrome C; FAD/FADH_2_—flavin adenine dinucleotide; MEL—melatonin; NAD/NADH—nicotinamide adenine dinucleotide; SIRT1—sirtuin 1 (silent mating type information regulation 2 homolog 1); PGC–1α—receptor gamma coactivator 1-alpha; UQ—ubiquinone; Δψm—mitochondrial membrane potential. This scheme was created using Servier Medical Art (available at https://smart.servier.com/) (accessed on 1 March 2025).

Notably, MEL shields cardiolipin, an essential phospholipid of the mitochondrial inner membrane, against oxidative insults, which contributes to preserving membrane structure and optimal electron transport chain performance. It also influences mitochondrial biogenesis by upregulating sirtuins, particularly SIRT1 and SIRT3, which play roles in deacetylating mitochondrial proteins and enhancing complex I activity [1,60]. MEL enhances SIRT3 expression and activity via the 5′ adenosine monophosphate-activated protein kinase (AMPK)–peroxisome proliferator-activated receptor-gamma coactivator 1-alpha (PGC-1α)–estrogen-related receptor alpha (ERRα) signaling axis. This pathway regulates mitochondrial biogenesis and promotes deacetylation of key antioxidant enzymes, including SOD2 and catalase [61,63]. PGC-1α acts not only as a mediator of mitochondrial fission regulation via dynamin-related protein 1 (Drp1) transcription but also as a master regulator of mitochondrial biogenesis through transcriptional coactivation of nuclear respiratory factors [63,64]. Recent findings also suggest that MEL prevents pathological mitochondrial fission by inhibiting the expression of the key fission proteins Drp1 and mitochondrial fission protein 1 (Fis1) via activation of the SIRT1–PGC-1α pathway (Scheme 5) [63]. Dong et al. [63] demonstrated that cadmium induces nephrotoxicity by upregulating Drp1- and Fis1-mediated mitochondrial fission in rat proximal tubular cells. MEL counteracted this effect by activating the SIRT1–PGC-1α axis, which suppressed the transcription and translation of both fission mediators. This intervention preserved mitochondrial structure and function, limited ROS production, and attenuated Cd-induced apoptosis. These findings support the concept that MEL modulates mitochondrial dynamics not only in neuronal or cardiovascular tissue but also in renal models of oxidative injury. In cadmium-induced proximal tubular cell injury, MEL restored mitochondrial morphology, suppressed ROS production, preserved membrane potential, and increased mitochondrial mass. Chromatin immunoprecipitation assays further confirmed that PGC-1α directly binds to the Drp1 promoter, linking melatonin’s mitochondrial protection to transcriptional regulation of fission machinery. These effects underscore melatonin’s broader ability to modulate mitochondrial dynamics and resist stress-induced fragmentation. The beneficial effects of melatonin and its metabolites, AFMK and AMK, on mitochondria include stimulation of mitochondrial biogenesis, promotion of fusion, enhancement of mitochondrial membrane potential, and increased ATP production.

In addition, SIRT1 appears to mediate MEL’s anti-inflammatory effects by inhibiting inflammatory pathways and preventing overactivation of nitric oxide synthase, thereby indirectly preserving mitochondrial function in inflammatory conditions [53]. Through these mechanisms, MEL is considered a key modulator of mitochondrial integrity and performance, especially in the face of neurodegenerative or oxidative stress conditions [1,60]. MEL has also been reported to modulate mitochondrial dynamics in neurodegenerative contexts beyond classical proteinopathies. In a prion disease model, MEL pretreatment was associated with prevention of mitochondrial fragmentation, preservation of Δψm, and reduced ROS overproduction. It also regulated Drp1 and optic atrophy 1 (OPA1) expression, potentially protecting neuronal cells from apoptosis and synaptic injury [65].

Further evidence of melatonin’s beneficial effects on mitochondrial complex I comes from clinical studies in PD. In a double-blind, randomized, placebo-controlled clinical trial conducted in patients with PD, Jiménez-Delgado et al. [66] demonstrated that oral supplementation with MEL significantly enhanced mitochondrial activity and bioenergetic capacity. The study showed that MEL administration increased the enzymatic activity of mitochondrial complex I, a key component of the electron transport chain responsible for the initial transfer of electrons from nicotinamide adenine dinucleotide (NADH) to coenzyme Q. Complex I dysfunction is associated with impaired oxidative phosphorylation and elevated production of ROS; MEL appears to counteract these effects by restoring complex I function and reducing markers of oxidative damage in plasma [66]. Moreover, MEL treatment significantly improved the respiratory control ratio (RCR), a widely recognized indicator of mitochondrial coupling efficiency, without altering mitochondrial membrane fluidity. These findings underscore melatonin’s role in preserving mitochondrial integrity and functionality, potentially through its antioxidant action and ability to modulate respiratory chain components expression or activity [66]. MEL has also been reported to provide protection in HD genetic models. In transgenic HD mice, MEL administration was associated with delayed disease onset and reduced mortality. Interestingly, mutant huntingtin (mHTT) was associated with a marked reduction of mitochondrial MT1 receptors, otherwise abundant in healthy brain mitochondria. MEL treatment appeared to inhibit mHTT-induced caspase activation and preserve MT1 receptor expression. Notably, this neuroprotective effect was dependent on the presence and activation of MT1, suggesting that mitochondrial MT1 signaling may play a pivotal role in neuronal resilience against HD-related toxicity [67]. A similar mitochondrial pathology is observed in retinal neurodegenerative diseases, particularly age-related macular degeneration (AMD), which shares several pathophysiological features with central nervous system diseases, including impaired mitochondrial biogenesis, elevated ROS production, and mtDNA damage. In AMD-affected retinal pigment epithelial (RPE) cells, studies have documented reduced Δψm and compromised respiratory chain function, especially in complexes I and III. A decrease in the expression of mitochondrial chaperones such as HSP60 and HSP70, critical for protein folding and mtDNA replication, has also been observed [61]. These findings further support the concept that mitochondrial instability plays a central role in oxidative stress-related degenerative diseases. In particular, damage to mtDNA regions encoding essential respiratory proteins and ribosomal RNAs appears to represent a key converging mechanism. Importantly, MEL has been shown to alleviate these changes by preserving Δψm, reducing oxidative damage to mtDNA, and stabilizing mitochondrial protein homeostasis, suggesting broader therapeutic applicability beyond neurodegeneration [61].

Beyond its antioxidant capacity, MEL exerts multifaceted effects on mitochondrial homeostasis. Owing to its amphiphilic nature, it accumulates in mitochondria, where it scavenges ROS and RNS and induces endogenous antioxidant enzymes, such as GPx, SOD, and glutathione reductase (GR) [58]. It also stabilizes mitochondrial membranes, preserves the activity of OXPHOS complexes, and modulates mitochondrial dynamics and apoptosis-regulating proteins, including family proteins Bcl-2 and Bax. Notably, melatonin’s protective actions are complemented by the activity of its oxidative metabolites, including AFMK and AMK. These compounds independently scavenge a wide range of reactive species (e.g., ^·^OH, O_2_^−^, H_2_O_2_, NO, and ONOO^−^) and penetrate into mitochondria, supporting redox homeostasis in both physiological and pathological contexts [60]. Interestingly, AFMK can also be formed within mitochondria via pseudoenzymatic oxidation of MEL by oxoferryl cytochrome c, a process that not only generates this protective metabolite but also helps restore the redox cycling of cytochrome c during oxidative stress [53,61]. In addition to its scavenging activity, AMK also limits the overproduction of nitric oxide by downregulating inducible and neuronal nitric oxide synthases inducible nitric oxide synthase (iNOS) and neuronal NOS (nNOS)). This suppression of NO synthesis reduces the formation of peroxynitrite and downstream toxic radicals, such as carbonate and nitrogen dioxide species, which are known to damage mitochondrial components and disrupt Δψm [53]. AMK in particular exerts potent detoxifying effects against reactive nitrogen species, notably nitric oxide and its toxic derivatives, including carbonate and nitrogen dioxide radicals. Unlike MEL, which can redonate NO under some conditions, AMK forms stable adducts, thereby neutralizing excess NO without re-release [53]. These actions may contribute to the preservation of Δψm and the inhibition of mPTP opening, though further research is warranted to clarify their direct impact on these parameters. By inhibiting NO accumulation and neutralizing its downstream derivatives, AMK protects mitochondria from peroxynitrite-mediated damage. Peroxynitrite, formed from NO and superoxide, contributes to mitochondrial dysfunction by initiating the formation of highly reactive radicals, including ^•^OH, CO_3_^•−^, and NO_2_, which target electron transport complexes and membrane lipids such as cardiolipin [53].

Furthermore, Reiter and colleagues highlight that in healthy cells, mitochondria can synthesize MEL autonomously, independent of the pineal gland. However, in pathological conditions, pyruvate dehydrogenase complex (PDH) activity is suppressed by pyruvate dehydrogenase kinase (PDK). As a result, pyruvate is diverted from the mitochondria, which impairs acetyl-CoA production and may halt mitochondrial MEL synthesis. This mechanism may underlie the mitochondrial dysfunction observed not only in cancer but also in other oxidative stress-associated diseases such as neurodegeneration, metabolic syndrome, ischemia–reperfusion injury, and sepsis. In such contexts, supplementation with exogenous MEL has been reported to attenuate mitochondrial damage and improve cellular bioenergetics, suggesting its therapeutic relevance [64].

Using an integrated multi-omics approach, Jiang et al. [68] demonstrated that chronic low-dose MEL supplementation effectively counteracts age-related mitochondrial dysfunction in the hippocampus. In their study, MEL was shown to regulate systemic and brain-specific energy metabolism in an age-dependent manner, with the most pronounced effects observed in middle-aged mice. Transcriptomic, proteomic, and lipidomic analyses revealed that MEL improved mitochondrial electron transport, restored mitochondrial morphology, and modulated key metabolic pathways such as valine, leucine, and isoleucine degradation. Importantly, lipidomic profiling showed that aging was associated with a significant upregulation of long-chain unsaturated glycerophospholipids in the hippocampus. These lipid species are highly susceptible to peroxidation and may contribute to mitochondrial damage. MEL effectively reversed these lipid alterations and reduced oxidative stress, likely by stabilizing mitochondrial membranes and regulating lipid-associated proteins such as Mpst, Ccsap, and Flna. Furthermore, the study identified that MEL enhanced antioxidant enzyme activity and suppressed stress-induced cytochrome C release via MEL receptors on the outer mitochondrial membrane [68]. In addition, MEL may protect mitochondrial DNA from oxidative damage by reducing the accumulation of 8-hydroxy-2′-deoxyguanosine (8-OHdG), a mutagenic base modification. By limiting 8-OHdG formation, MEL may help to prevent mtDNA mutations and support mitochondrial genome stability, particularly in aging or stress-exposed tissues [61]. These findings support the hypothesis that MEL may protect against mitochondrial dysregulation and mitochondrial deterioration through multi-layered mechanisms involving lipid homeostasis, antioxidant defense, and mitochondrial proteostasis. Collectively, these data suggest that MEL may have therapeutic potential in delaying or reversing mitochondrial aging, particularly in the brain [68].

Beyond pathological states, the potential of MEL to counteract physiological mitochondrial decline during aging is gaining attention. Recent multi-omics studies provide evidence for its potential anti-aging mitochondrial effects, especially in the hippocampus. Given its amphiphilic nature and subcellular accumulation in mitochondria, MEL emerges as an ideal mitochondrial antioxidant [58,60]. By combining direct scavenging actions with modulation of mitochondrial enzymes and membrane structure, MEL is pivotal in preserving bioenergetic homeostasis across various pathological settings.

## 5. Melatonin and Its Metabolites—Reduction of Metal Toxicity

One of the proposed antioxidant mechanisms by which melatonin may contribute to neuroprotection involves the modulation of redox-active metal toxicity in the central nervous system. Although trace metals such as iron and copper are essential for numerous physiological processes, their accumulation or dysregulation can lead to the generation of ROS, contributing to oxidative damage, lipid peroxidation, and impairment of both enzymatic and non-enzymatic antioxidant defenses [69].

**Scheme 6:**
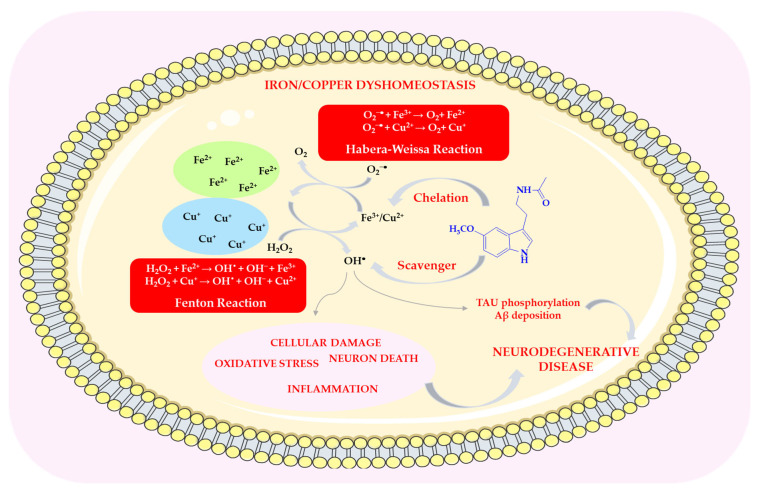
The role of melatonin in reducing metal toxicity. This scheme was created using Servier Medical Art (available at https://smart.servier.com/) (accessed on 1 March 2025).

Melatonin prevents metal oxidation by neutralizing free radicals produced in redox reactions, such as the Fenton reaction and the Haber–Weiss reaction (HWR), or by chelating metal ions [70]. In both redox reactions, two metal ions are most commonly involved (Fe(II) and Cu(II)—see Scheme 6). By chelating copper and iron ions, melatonin removes them from both intra- and extracellular environments, thereby inhibiting metal-dependent production of free radicals [71].

In the Fenton reaction, Fe^2+^ is oxidized by H_2_O_2_ to Fe^3+^, which is then reduced back to Fe^2+^ at the expense of endogenous reducing agents, for example, in the Haber–Weiss reaction with the superoxide anion radical. The Fenton reaction is the primary mechanism for generating hydroxyl radicals under physiological conditions [70,72]. Iron overload can lead to abnormalities that affect the functions of cells or tissues. Melatonin is capable of interacting with iron(III) ions, removing free Fe^3+^ and thereby preventing its reduction to Fe(II) and avoiding the generation of free radicals [70,73]. It inhibits iron-dependent cell death, known as ferroptosis, which leads to damage and disorganization of the cell membrane [71,74]. Its derivative, 6-OHM, reduces Fe^2+^-induced neurotoxicity [75]. For this reason, MEL and its metabolites may serve as effective therapeutic agents in iron-induced oxidative stress, for example, in patients with Parkinson’s and Alzheimer’s disease, in whom higher levels of iron and lipid peroxidation have been observed in the brain compared to healthy individuals [76,77]. Administration of melatonin at pharmacological doses (5 mg/kg body weight per day) following intracerebral iron injection reduced neuronal death by approximately 40% [70,78].

Copper, like iron, can participate in redox cycling, producing ROS directly or indirectly by reducing to Cu^+^ and reacting with hydrogen peroxide to form hydroxyl radicals [70,79].

Copper overload, similar to iron overload, leads to impaired cell function and eventually cell death. An imbalance between copper absorption and excretion may be associated with neurodegenerative diseases, including AD, PD, and HD [80,81,82,83,84]. MEL and its metabolites (AFMK, AMK, and c3-OHM) can chelate Cu(II), preventing its reduction and subsequent hydroxyl radical production via Fenton-like reactions (Scheme 6) [5,69,85,86].

In addition to iron and copper, melatonin has shown protective properties against the neurotoxic effects of other metals, including cadmium, mercury, arsenic, lead, aluminum, nickel, and cobalt [70,87,88,89,90,91,92]. Mercury and cobalt may be involved in some neurodegenerative disorders, such as Alzheimer’s disease [70,93]. On the other hand, high levels of aluminum in the brain are likely associated with, among other things, AD and PD [70].

Overall, the available preclinical data suggest that melatonin and its metabolites may reduce metal-induced oxidative stress through both chelation and radical-scavenging mechanisms. These findings highlight the need for further investigation into their potential utility as adjunctive agents in conditions characterized by metal dysregulation and oxidative stress.

## 6. Melatonin in Alzheimer’s Disease

**Scheme 7:**
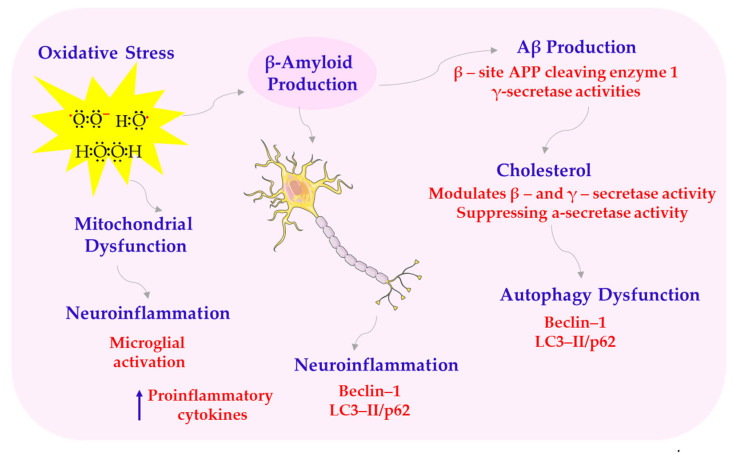
Structural abnormalities of AD. APP—amyloid precursor protein; LC3-II/p62—microtubule-associated protein 1A/1B-light chain 3. This scheme was created using Servier Medical Art (available at https://smart.servier.com/) (accessed on 1 March 2025).

Alzheimer’s disease is the most common form of dementia, accounting for 60–80% of cases. It is characterized by progressive cognitive and memory impairment, primarily due to β-amyloid (Aβ) deposition and tau hyperphosphorylation, leading to synaptic dysfunction and neurodegeneration. Recently, increasing attention has been paid to additional contributors to AD pathogenesis. These include oxidative stress, metabolic disturbances, cerebrovascular dysfunction, and abnormal activation of glial cells. Given the growing prevalence of AD and the lack of effective treatments, understanding its complex pathogenesis is crucial for developing new therapeutic strategies (see Scheme 7) [94].

One key mechanism of melatonin’s neuroprotective effect is its influence on amyloid precursor protein (APP) metabolism. It modulates enzymes involved in APP processing, promoting non-amyloidogenic pathways and reducing the production of toxic amyloid-β peptides. On the one hand, melatonin increases the expression of α-secretase, a disintegrin and metalloproteinase domain-containing protein 10 (ADAM10), which results in the production of neuroprotective sAPPα. On the other hand, it inhibits the expression of β-secretase β-site APP cleaving enzyme 1 (BACE1) and affects the activity of the γ-secretase complex, which is a component of presenilin-1 (PS1), limiting the formation of neurotoxic Aβ1-42 [65,95,96,97,98]. Another important mechanism involves melatonin’s role in supporting mitochondrial function and reducing oxidative stress. It stabilizes cardiolipin and respiratory chain enzymes, reduces the production of ROS, and limits the formation of pathological mitochondrial supercomplexes. In conditions of oxidative stress, melatonin reduces the expression of activated caspase-3 and the Bax/Bcl-2 ratio, preventing neuronal apoptosis [50,99,100,101]. Another key effect of melatonin is its influence on circadian rhythms and sleep quality. Sleep disturbances are common in AD, and melatonin supplementation improves sleep duration, increases rapid eye movement (REM) phase length, and reduces nighttime awakenings. These improvements also contribute to better cognitive performance and reduced neuropsychiatric symptoms [102,103,104]. Moreover, low levels of melatonin in AD patients correlate with the severity of cognitive symptoms and behavioral disorders [105].

Melatonin stabilizes the expression of clock genes such as brain and muscle ARNT-like 1 (BMAL1) and period circadian protein homolog 2 (PER2), restoring neuronal rhythmicity and improving the synchronization of brain structures regulating sleep and wakefulness [106,107]. Improved sleep may also reinforce melatonin’s broader neuroprotective effects.

In addition, melatonin regulates the expression of Bcl-2, inhibits caspase activity, and supports the function of the BBB, limiting the penetration of inflammatory cells and harmful metabolites into the brain. It may also exert beneficial effects on glucose and lipid metabolism, alleviating inflammatory processes and improving neuronal survival [108,109].

In its immunomodulatory aspect, melatonin limits the accumulation of inflammatory cells, inhibits the activity of nicotinamide adenine dinucleotide phosphate oxidase (NADPH oxidase), and supports the activity of anti-inflammatory cytokines, such as interleukin-10 (IL-10) and neurotrophins [110,111].

In the context of metabolic disorders, studies suggest that melatonin restores the activity of the PI3K/AKT/mechanistic target of rapamycin (mTOR) pathway, reduces insulin receptor substrate-1 (IRS-1) phosphorylation, and increases insulin-like growth factor 1 (IGF-1) activity. This results in improved glucose transport, increased ATP availability, and reduced oxidative stress in conditions of insulin resistance [112,113,114,115]. In addition, increased expression of 5-lipoxygenase-activating protein (5-LOX) and 12/15-LOX, enzymes that strongly induce Aβ production and promote inflammation, is observed in the brains of AD patients [116,117]. Overexpression of 5-LOX may be partially related to melatonin deficiency in the elderly. Studies have shown that even at nanomolar concentrations, melatonin inhibits 5-LOX gene expression by acting on high-affinity nuclear melatonin receptors. Suppression of this pathway reduces the response inflammatory process and reduces oxidative stress, which translates into reduced neuronal damage and Aβ-induced neurotoxicity [101].

Melatonin also affects key transcriptional pathways: SIRT1/forkhead box protein O (FOXO), Nrf2/ARE, the canonical Wnt pathway (Wnt/β-catenin), and MAPK/ERK. By activating these pathways, it supports neuronal differentiation, limits apoptosis, and promotes the regeneration of neural tissues [101,109,118,119].

Stabilization of calcium metabolism is another important mechanism of melatonin’s action. By inhibiting the excessive activation of N-methyl-D-aspartate receptor (NMDAR), binding to calmodulin, and regulating calcium-dependent kinases such as Ca^2+^/calmodulin-dependent protein kinase II (CaMKII) and PKC, melatonin prevents excitotoxicity and activation of calcium-dependent caspases. It can also affect calreticulin, stabilizing APP function [101,120]. In animal models in which amnesia was induced by scopolamine, an antagonist of muscarinic acetylcholine receptors, melatonin was shown to significantly improve cognitive functions and restore the activity of the cholinergic system in the hippocampus and medial septum. This mechanism involves increased expression and immunoreactivity of key proteins involved in the cholinergic neurotransmission cycle: acetylcholine transferase (ChAT), high-affinity choline transporter (CHT), vesicular acetylcholine transporter (VAChT), and muscarinic M1 receptor (M1R). Melatonin not only increases their protein levels but also restores the morphological structure and density of cholinergic fibers in the hippocampus, which translates into improved cognitive performance in experimental animals [121]. Moreover, there are data indicating that melatonin can act as an acetylcholinesterase (AChE) inhibitor, which further enhances cholinergic transmission by prolonging the availability of acetylcholine in the synaptic cleft [122]. This action may also be associated with the regulation of intracellular calcium levels, which play an important role in the release of neurotransmitters. Therefore, melatonin appears to be an important factor supporting neuroprotection in Alzheimer’s disease, through multidirectional modulation of cholinergic system function, which may be crucial for alleviating cognitive symptoms in patients with AD.

One of the most promising areas of melatonin’s action is its effect on neurogenesis and synaptic plasticity. Melatonin increases the proliferation of progenitor cells, promotes their differentiation towards neurons, and supports their functional integration. It acts through the Wnt, Notch, PI3K/AKT pathways and stimulation of neurotrophic factors (such as BDNF and nerve growth factor (NGF)) and the transcription factor CREB [118,123,124].

Additionally, studies suggest that melatonin promotes mitochondrial repair and morphology, improves redox, and further reduces oxidative stress markers [125,126].

In models of aging and AD, long-term administration of melatonin appears to inhibit neurodegeneration, improve cognitive function, reduce amyloid deposits and tau phosphorylation, and reduce microglial activation and inflammatory cytokines [127,128,129,130].

In light of the above data, melatonin shows potential as a multitarget agent, capable of modulating multiple pathways of Alzheimer’s disease pathomechanisms at the molecular, cellular, and functional levels. Its multidirectional action makes melatonin a valuable candidate for preventing and supporting the treatment of age-related neurodegeneration and AD.

## 7. Melatonin in Parkinson’s Disease

**Scheme 8:**
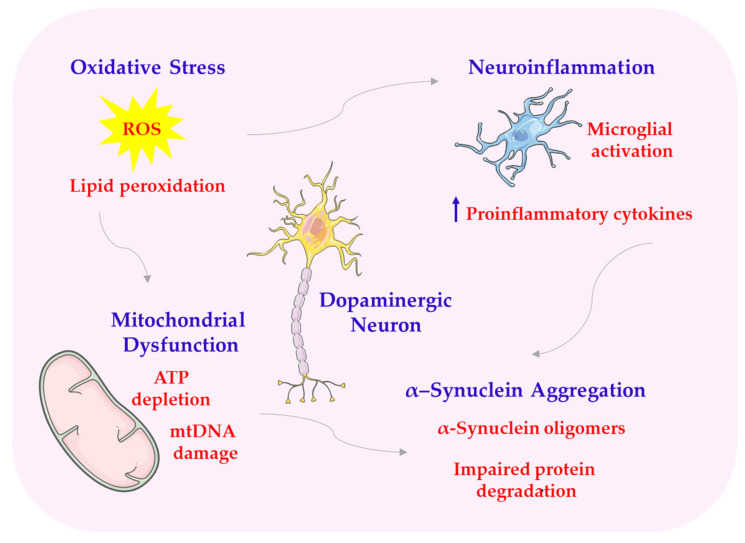
Structural abnormalities of PD. ATP—adenosine triphosphate; mtDNA—mitochondrial DNA. This scheme was created using Servier Medical Art (available at https://smart.servier.com/) (accessed on 1 March 2025).

Parkinson’s disease is the most common neurodegenerative movement disorder, affecting about 1% of the population over 60 years of age. Its etiology remains unknown in most cases, although a genetic basis of the disease is identified in about 5–15% of patients. Parkinson’s disease is marked by the loss of dopamine-producing neurons in a part of the brain called the substantia nigra and the buildup of incorrectly folded α-synuclein proteins, which form structures known as Lewy bodies. The pathogenetic mechanisms in Parkinson’s disease are highly complex. They include mitochondrial dysfunction, oxidative stress, autophagy disorders, protein aggregation, and neurogenic inflammation. This complex poses a significant challenge in developing disease-modifying therapies [131] (see Scheme 8).

Melatonin exerts antioxidant effects, in part by activating the Nrf2/ARE pathway. This pathway regulates the expression of antioxidant and detoxifying enzymes [5,132,133]. Melatonin accumulates in mitochondria, the main source of ROS, where it stabilizes membrane potential, prevents excessive opening of mPTP, limits the release of cytochrome c, and supports the functioning of the respiratory chain (complexes I-IV). Thanks to these properties, melatonin improves the bioenergetic efficiency of neurons, increases ATP synthesis, and limits oxidative damage to mtDNA [1,134]. Studies on animal models of PD, such as MPTP-treated mice or 6-OHDA-injected rats, suggest that melatonin administration may reduce oxidative stress markers in the substantia nigra and striatum and improve the survival of dopaminergic neurons [58]. In in vitro studies, melatonin protected SH-SY5Y cells from rotenone- or neurotoxin-induced toxicity associated with inhibiting mitochondrial complex I [135]. The ability of melatonin to affect SIRT1/SIRT3 may therefore be one of the key mechanisms of its action in the protection of dopaminergic neurons in the course of Parkinson’s disease [136]. Melatonin may increase SIRT1 activity, which leads to the activation of the transcription factor FOXO3a, responsible for the expression of antioxidant enzymes and proteins involved in DNA repair. In PD models, activation of this pathway by melatonin has been shown to improve the survival of dopaminergic neurons and reduce motor symptoms [66,137,138]. Melatonin’s action also includes inhibition of the intrinsic apoptosis pathway by regulating the expression of Bcl-2 family proteins. In particular, melatonin increases the expression of Bcl-2, a protein with antiapoptotic properties, while reducing the level of Bax—a protein promoting apoptosis—and inhibiting the activity of caspase-9 and caspase-3. The reduction in the Bax/Bcl-2 ratio is one of the most important indicators of the neuroprotective effect of melatonin under conditions of oxidative stress and environmental toxicity [119,139]. At the same time, melatonin affects the second important process, autophagy, which is disturbed in the course of PD. Autophagy is responsible for the removal of damaged organelles, excess proteins, and aggregates, including toxic α-synuclein. Properly functioning autophagy prevents the accumulation of abnormal cellular structures and counteracts cell death. However, in Parkinson’s disease, impaired autophagy is observed, which contributes to neurodegeneration [4,139,140]. Melatonin modulates autophagy by inhibiting mTOR and activating Beclin-1, which is a key element initiating the autophagy process. Melatonin inhibition of mTOR enables autophagy to be triggered, which leads to increased clearance of damaged mitochondria (mitophagy) and improved cell function [141,142]. In addition, melatonin promotes the expression of LC3-II, a marker of mature autophagosomes, and reduces the level of p62, the accumulation of which is characteristic of impaired autophagy [143]. Melatonin induces the translocation of Nrf2 to the cell nucleus and the activation of ARE, which may protect neurons from the toxic effects of free radicals and metal catalysts of redox reactions, such as iron [144,145]. Melatonin also affects the Wnt/β-catenin pathway, which plays an important role in regulating the proliferation, differentiation, and survival of nerve cells. Activation of this pathway supports the process of neurogenesis, synaptic plasticity, and repair of damaged neural tissues. In the context of PD, melatonin has been shown to counteract the inhibition of the Wnt pathway induced by oxidative and inflammatory stress, thereby promoting the survival of dopaminergic neurons [118]. The effect of melatonin on the expression of the BMAL1 protein, a fundamental molecular component of the biological clock, also plays an important role. BMAL1 expression is reduced in the brains of PD patients and is associated with the severity of symptoms and the disruption of metabolic and immune homeostasis. Melatonin supports the restoration of rhythmic BMAL1 expression, which indirectly affects redox balance, regulation of inflammatory processes, and physiological rhythms in the central nervous system (CNS) [146].

Studies have shown that melatonin can also affect proteins responsible for protein quality control (e.g., chaperones, ubiquitination), which supports the degradation of improperly folded proteins and reduces the risk of aggregate formation. Due to antiapoptotic and autophagy-promoting effects, melatonin may protect neurons from death. It may also improve their adaptive and regenerative capacity under neurotoxic conditions [60,147].

Melatonin may exert an anti-aggregation effect on α-synuclein. It acts directly by binding to this protein and stabilizing its conformation and indirectly by supporting degradation mechanisms through activation of autophagy and the ubiquitin-proteasome system. By activating the Beclin-1 pathway and inhibiting mTOR, melatonin improves the clearance of pathological forms of α-synuclein, which limits their toxic effects on nerve cells [148,149,150,151,152]. In addition, melatonin plays an important role in preventing another type of cell death associated with PD, which is ferroptosis. Ferroptosis is a form of programmed cell death that is iron-dependent and is characterized by the accumulation of lipid peroxides and a decrease in glutathione levels. Ferroptosis is particularly important in dopaminergic neurons due to their high iron content and susceptibility to lipid peroxidation [153]. Melatonin may counteract ferroptosis. It activates the MT1/SIRT1/Nrf2 pathway, increasing expression of heme oxygenase-1 (HO-1) and GPx, which protect against lipid peroxidation. In parallel, melatonin maintains glutathione levels and prevents the accumulation of free iron in the cytoplasm by regulating the expression of ferritin and ferroportin transporter proteins. As a result, it limits the formation of free hydroxyl radicals in Fenton reactions [153,154]. Experimental studies have shown that melatonin reduces the expression of the ferroptosis marker ACSL4 and limits oxidative lipid damage in dopaminergic neurons treated with neurotoxins. In vivo models have been shown to reduce substantia nigra degeneration and improve motor function after melatonin supplementation, confirming its efficacy in inhibiting ferroptosis [153,155]. Melatonin may exert anti-inflammatory effects at both the cellular and molecular levels. It inhibits microglial activation and the expression of activation markers such as Iba-1 and CD11b [156]. Furthermore, melatonin reduces the expression of pro-inflammatory genes by blocking the toll-like receptor 4 (TLR4)/myeloid differentiation primary response 88 (MyD88)/NF-κB signaling pathway, which plays a key role in inflammatory signal transduction in glial cells. By blocking the activation of the transcription factor NF-κB, melatonin limits the transcription of genes encoding cytokines, chemokines, and pro-inflammatory enzymes (e.g., cyclooxygenase-2 (COX-2), iNOS) [157,158]. In addition, melatonin inhibits the activation of the NLRP3 inflammasome, a molecular complex responsible for the activation of caspase-1 and the maturation of interleukin-1β (IL-1β) and interleukin-18 (IL-18). By lowering ROS levels and stabilizing mitochondrial function, melatonin prevents inflammasome activation. This action helps to limit inflammation-induced neuronal damage [159,160].

Experimental studies have shown that melatonin restores the rhythmicity of BMAL1 and PER2 expression and improves neuronal synchronization in the SCN [161,162].

Clinically, melatonin supplementation has been associated with improved sleep quality, reduced RBD episodes, increased total sleep time, and reduced number of awakenings. Melatonin also improves subjective sleep quality parameters assessed on scales such as the Pittsburgh Sleep Quality Index (PSQI). In addition, the sleep-improving effect of melatonin contributes to the reduction of daytime symptoms outside of sleep; motor symptoms such as fatigue; depression; irritability; and cognitive impairment [163,164]. Melatonin also affects peripheral biological clocks, including those in the liver, intestines, and heart, which may be important in the context of the brain–gut axis and the links between microbiota and neurodegeneration. Stabilization of circadian rhythms in these organs may indirectly support neuroimmune and metabolic homeostasis, contributing to the overall improvement of the condition of patients with Parkinson’s disease [165].

## 8. Melatonin in Huntington’s Disease

Huntington’s disease is an autosomal dominant neurodegenerative disorder. It primarily affects patients between the ages of 30 and 50, and despite extensive research, no effective treatment has been found so far. It is a progressive and ultimately fatal disease. HD is characterized by involuntary choreic movements along with cognitive and behavioral disturbances. Symptoms and signs classically include motor, cognitive, and psychiatric disorders [4,166]. Other less common symptoms include weight loss, sleep disturbances, and autonomic nervous system dysfunction [4]. The disease is characterized by overall brain atrophy and degeneration of the striatum (caudate nucleus and putamen), and it primarily targets GABAergic medium spiny neurons (MSNs) in the striatum [167].

In the early stages of the disease, cortical mass loss is observed, progressing from the posterior to the anterior cortical regions. HD is caused by mHtt, a protein with a molecular weight of 350 kDa, which is the primary cause of the disease [58,166,168]. The genetic mutation in HD is characterized by an expansion of cytosine–adenine–guanine (CAG) repeats within the exon region of the HTT gene [166]. It occurs on the short arm of chromosome 4p16.3. This leads to a long expansion of the polyglutamine (poly-Q) tail in the HTT protein through uninterrupted trinucleotide CAG repeats [168]. The length of the CAG repeats normally ranges from 11 to 29, but in patients with HD, there are more than 40 repeats. This poly-Q tail undergoes cleavage by the proteasome, generating protein fragments prone to abnormal aggregation and accumulation with neurotoxic properties.

The aggregation of the mutated HTT gene triggers neuronal cell death through the apoptotic pathway, which mainly results from mitochondrial dysfunction [166,169]—one of the important mechanisms involved in the pathology of HD [2,23,170].

A secondary event following the gene mutation is the impairment of the electron transport chain [1], resulting in defects in ATP production.

In HD, there is a deficiency of respiratory chain complex II, III, and IV components in the caudate nuclei and putamen of patients. The preceding event involves abnormal ATP production and defective calcium processing in the mitochondria. An increase in Ca^2+^ levels makes the neuron more susceptible to excitotoxicity, a process arising from changes in neurotransmitter release [1,171].

Excitotoxicity is caused by a combination of increased glutamate release and glutamate agonists from the afferent fibers of the cerebral cortex [168]. Excitotoxicity in HD is likely triggered by a tryptophan metabolite, quinolinic acid, which is considered a factor causing neuropathology. It has been observed in the brains of individuals who died from Huntington’s disease. This metabolite, being a strong neurotoxin, acts on NMDA receptors on postsynaptic neurons and consequently leads to the generation of ROS. This causes molecular changes within the cells and ultimately leads to their death [23]. Additionally, it has been shown that mutated HTT induces mitochondrial fragmentation through direct association with the mitochondrial membrane and by increasing the activity of Drp1 [166]. Excessive mitochondrial division triggered by mutated HTT may play an important role in the striatum’s susceptibility to the disease [166,172].

**Scheme 9:**
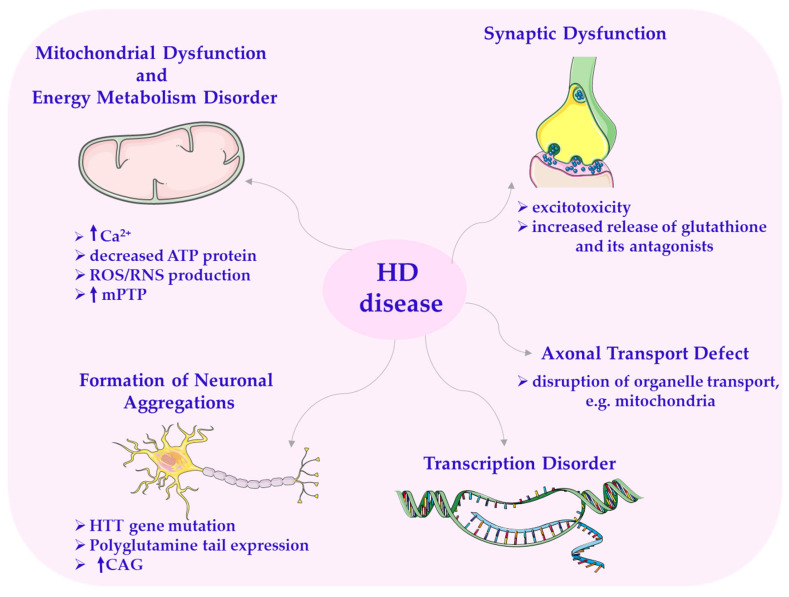
Disorders in Huntington’s disease. CAG—cytosine–adenine–guanine; HTT—huntingtin gene. This scheme was created using Servier Medical Art (available at https://smart.servier.com/) (accessed on 1 March 2025).

It has also been proven that aberrant mitochondrial division causes pathological changes in HD. It leads to increased oxidative stress, alterations in mitochondrial bioenergetics, or impaired mitochondrial transport to synapses [1,2,166]. In HD, there can also be disturbances in transcription and synaptic dysfunction [168], as well as deletions in mtDNA (mtDNA4977), particularly in mitochondria from the frontal and temporal lobes. Increased production of ROS/RNS generated by mitochondria and the accumulation of mtDNA mutations are factors contributing to neurodegeneration in HD (Scheme 9) [166].

In recent years, more attention has been paid to studies evaluating the effect of the pineal hormone melatonin on mitochondrial dysfunction, as an important factor contributing to, among other things, neurodegenerative disorders [4,166].

Based on numerous experimental animal studies, it has been documented that this hormone may have the potential to protect nerve cells from the action of the neurotoxin 3-nitropropionic acid. It inhibits not only oxidative stress but also neuronal damage caused by neurotoxins, inducing HD, both in vitro and in vivo [173,174,175]. These studies have shown and confirmed that melatonin prevents lipid peroxidation and protein carbonylation and influences the balance between pro- and antioxidant effects. It has been proven that it can be used to modify the neural response to 3-nitropropionic acid, and its protective mechanism is associated with antioxidant properties [2].

Melatonin also acts in another molecular mechanism of cell death dependent on mitochondria, which is associated with the elevation of intracellular Ca^2+^ concentration [171,176]. It has been documented that melatonin reduces the increase in Ca^2+^ concentration induced by the N-methyl-D-aspartate receptor by inhibiting the activity of mPTP in primary striatal neurons in mice [177]. Furthermore, melatonin exhibits a protective effect on neuronal apoptosis by regulating pro- and/or anti-apoptotic proteins [178], which is related to its ability to eliminate free radicals.

Preclinical studies in a transgenic R6/2 HD mouse model using striatal cells with the HTT ST14A mutation showed that melatonin effectively inhibited not only cytochrome c release but also cell death. It also prevented the loss of the MT1 receptor after injury [179], which may help to limit mitochondrial damage in HD through the MT1 receptor-dependent pathway.

In another report, Wang et al. demonstrated that melatonin delays disease progression and mortality in a transgenic mouse model of HD. They proved that it inhibits caspase activation induced by mutated HTT and preserves the expression of the mitochondrial receptor MT1. Its loss in HD not only increases neuronal susceptibility to damage but also accelerates the neurodegenerative process. It was found that this hormone counteracts MT1 depletion in both in vitro and in vivo conditions [67].

Of additional interest is the observation that plasma melatonin levels are reduced in patients with HD [179], raising the possibility that this hormonal deficiency may contribute to disease progression or symptom severity. Although preliminary, this observation suggests that melatonin supplementation warrants further exploration as a supportive intervention in HD and related disorders.

## 9. Therapeutic Efficacy of Melatonin in Neurodegenerative Diseases

Despite its promising neuroprotective properties, the therapeutic efficacy of melatonin is limited due to its low oral bioavailability (approximately 2.5%), poor absorption through mucosal membranes and the skin, low water solubility, relatively short half-life (30–60 min), and extensive hepatic metabolism [180,181].

Although melatonin can cross the BBB due to its amphiphilic nature, its concentration in the central nervous system after exogenous administration remains low. Therefore, conventional drug delivery offers limited therapeutic efficacy [182].

Biocompatible and safe nanocarriers used for CNS melatonin delivery include lipid-based, polymer-based, and hybrid (lipid–polymer) nanoparticles. For example, melatonin administration using lipid-based carriers such as liposomes and nanoemulsions has demonstrated a superior pharmacokinetic profile compared to melatonin dissolved in DMSO. Experimental studies show that intranasal administration of melatonin using lipid- and polymer-based nanocarriers extends its half-life in the brain and significantly enhances its bioavailability. This intranasal route bypasses the BBB via the olfactory and trigeminal nerves, enabling direct brain delivery [183,184].

Biomimetic nanoparticle platforms, designed to mimic natural biological structures and processes, are also being employed in the treatment of neurological disorders. Cell membrane-coated nanoparticles, for instance, have demonstrated potential in targeting the brain and treating conditions such as Parkinson’s disease and other disorders involving mitochondrial dysfunction [185].

Furthermore, human serum albumin nanoparticles containing melatonin have been shown to enhance mitophagy and mitochondrial biogenesis, thereby contributing to the prevention of Parkinson’s disease progression [186].

Combination therapies also represent a promising approach in slowing the progression of neurodegenerative diseases. For instance, a melatonin–alkylbenzylamine hybrid molecule has shown multifunctional activity in Alzheimer’s disease, including antioxidant properties and cholinesterase inhibition [187].

Lyotropic lipid-based liquid crystalline nanoparticles have attracted attention in nanomedicine for their ability to encapsulate therapeutic agents. These systems can transport lipophilic drugs directly from the nasal cavity to the brain, making them a promising strategy for melatonin delivery in the treatment of neurological diseases [188].

Although nanomedicine significantly improves melatonin’s bioavailability in the CNS, further pharmacokinetic, pharmacodynamic, and clinical studies (with therapeutic doses above 10 mg per day) are necessary to ensure long-term safety. Nonetheless, alternative delivery methods for melatonin present an important avenue for preventing and treating neurological disorders, requiring additional clinical validation to confirm their full therapeutic potential.

## 10. Melatonin Effects by Domain

This review comprehensively explores the therapeutic and functional significance of melatonin in the context of various neurodegenerative disorders, with a particular focus on Alzheimer’s disease, Parkinson’s disease, and Huntington’s disease. Melatonin, a neurohormone primarily secreted by the pineal gland, has been shown to exert multifaceted neuroprotective effects. Its mechanisms of action include the regulation of circadian rhythms, which are frequently disrupted in neurodegenerative diseases, thereby contributing to sleep disturbances and cognitive decline. Additionally, melatonin acts as a potent antioxidant, effectively scavenging reactive oxygen species and reducing oxidative stress, a key contributor to neurodegeneration. Furthermore, melatonin supports neuronal survival by preserving mitochondrial function, which is essential for cellular energy metabolism and apoptosis regulation. Moreover, melatonin influences key intracellular signaling pathways and gene expression patterns associated with inflammation, apoptosis, and neuronal plasticity. MEL modulates the expression of neurotrophic factors, regulatory enzymes, and structural proteins critical for maintaining neuronal integrity and function.

The diverse biological roles of melatonin in neurodegenerative diseases are summarized in Table 1, which outlines its impact on molecular pathways, cellular structures, and physiological processes across both experimental and clinical models.

## 11. Conclusions

This review highlights melatonin as a natural antioxidant with multiple biological effects. It plays a key role in redox regulation, mitochondrial function, and inflammatory signaling. Preclinical and early clinical studies show that melatonin can help maintain mitochondrial integrity, reduce oxidative damage, and support redox balance through direct and indirect actions. Melatonin acts as a free radical scavenger. It also boosts the effects of other antioxidants, such as vitamins C and E. These properties have been observed in experimental models of neurodegenerative and age-related diseases.

Clinically, melatonin is mostly used to treat sleep and circadian rhythm disorders. Sleep itself may affect oxidative stress and immune balance, especially in older adults and patients with neurodegeneration. Therapeutic doses typically range from 1 to 5 mg, but dosage should be tailored to the individual. Most studies involve small patient groups, limiting their reliability. However, melatonin is well tolerated for both short- and long-term use, with few side effects reported.

Further clinical trials are needed. These should define optimal dosing, improve bioavailability through new delivery methods, and assess long-term benefits. Larger, well-designed studies with higher doses and longer treatment periods are essential, especially in neurodegenerative disorders. Melatonin’s low toxicity and favorable safety profile make it a promising candidate for future therapies. Innovative approaches, such as nanocarriers, may enhance its pharmacokinetics and clinical utility. Given the rise in neurodegenerative diseases among aging populations, melatonin offers a safe and accessible option that warrants deeper investigation.

## Figures and Tables

**Table 1:** The role of MEL and its metabolites in various biological areas.

Biological Role of MEL and Its Metabolites		Mechanism of Action	References
Antioxidant		MEL and its metabolites neutralize a wide range of ROS and RNS, including superoxide anions, hydroxyl radicals, singlet oxygen, the oxoferryl radical of hemoglobin, peroxyl radicals, the carbonate radical, nitric oxide, and peroxynitrite anions. They also act against non-radical oxidants such as hydrogen peroxide and hypochlorous acid. Furthermore, MEL, AFMK, AMK, and c3-OHM are capable of scavenging the cationic radical 2,2′-azino-bis(3-ethylbenzthiazoline-6-sulfonic acid).	[12,13,14,15,16,17,18,19,20,21,22,23,24,25,26,27,31,32,34,35,36,37,38,39]
		MEL increases the production of important antioxidant enzymes in various tissues, such as GPx, CAT and SOD. MEL enhances the production of key antioxidant enzymes, including GPx, CAT, and SOD, across various tissues.	[43,44,45]
		MEL activates PKA, which in turn stimulates the transcription factor CREB. Activation of CREB leads to the upregulation of BDNF and Nrf2, further increasing the activity of SOD and GPx.	[14,41,46,47]
		MEL activates PI3K, which subsequently triggers the AKT and MAPK signaling pathways. Activation of the AKT pathway promotes SIRT3 activity, leading to increased glycolysis and reduced oxidative phosphorylation. Meanwhile, activation of the MAPK pathway contributes to the reduction of oxidative stress.	[46,49]
		MEL activates PLC, leading to an increase in intracellular calcium concentrations. Additionally, MEL binds to calmodulin, thereby modulating the synthesis of nitric oxide.	[49]
Mitochondrial Function		MEL and its metabolites (AFMK, AMK) stimulate mitochondrial biogenesis (via SIRT1/PGC-1α pathway), enhance mitochondrial fusion and membrane potential, suppress excessive mitochondrial fission, and increase ATP production.	[61,63,66,68]
		MEL preserves mitochondrial membrane potential (Δψm), inhibits the opening of the mitochondrial permeability transition pore (mPTP), and limits electron leakage, thereby reducing ROS formation and improving mitochondrial efficiency.	[1,3,60,62,66]
		MEL upregulates mitochondrial sirtuins (SIRT1, SIRT3) and activates the AMPK–PGC-1α–ERRα signaling pathway, leading to improved mitochondrial function and antioxidant defense	[1,5,61,63,68]
		MEL and its metabolites protect mitochondrial DNA from oxidative damage and preserve mitochondrial protein homeostasis, supporting genome stability and bioenergetics during stress and aging.	[61,68]
		MEL inhibits Drp1 and Fis1, key mediators of mitochondrial fission, maintaining a balance in mitochondrial dynamics.	[61,63]
Metal Toxicity		MEL chelates free Fe(III), preventing its reduction to Fe(II) and thereby inhibiting the Fenton reaction and subsequent free radical generation. Additionally, MEL forms complexes with aluminum, cadmium, and lead.	[73]
		MEL inhibits ferroptosis, a process that leads to cell membrane damage and disorganization. Mechanistically, MEL suppresses inflammatory responses and ferroptosis through activation of the MT2 and IL-33 pathways.	[74]
		MEL and 6-OHM have been shown to attenuate Fe^2+^-induced lipid peroxidation and necrotic cell damage in the rat hippocampus in vivo. A unilateral intrahippocampal infusion of a mixture containing 1 μL of iron citrate solution (Fe^2+^, 3.4 mmol/L) and 0.25 mL of either melatonin or 6-OHM at a dose of 10 mg/kg/day effectively alleviates Fe^2^-induced neurotoxicity in this brain region.	[75]
		Systemic administration of MEL (5 mg/kg body weight daily) resulted in a 40% reduction (*p* = 0.019) in neuronal death following intraventricular injection of 1.0 μL of 1.0 mM ferric ammonium citrate in rats. These findings indicate that pharmacological concentrations of melatonin effectively protect neurons against iron-induced damage.	[78]
		MEL and its metabolites (AFMK, AMK, c3-OHM) effectively inhibit oxidative stress induced by Cu(II)-ascorbate mixtures through chelation of Cu(II). Similarly, melatonin, AFMK, and 3-OHM can block the initial step of the Haber–Weiss reaction, thereby preventing hydroxyl radical formation via the Fenton reaction.	[85]
		MEL at a dose of 5 mg/kg body weight administered intraperitoneally exhibited neuroprotective effects in rats exposed to mercuric chloride. It significantly attenuated mercury-induced oxidative stress and structural brain damage caused by both low (2 mg/kg) and high (4 mg/kg) doses of mercuric chloride over a 60-day period.	[88]
		MEL at 4 or 20 mM protects against sodium arsenite (30 μM)-induced neurotoxicity in dorsal root ganglion explants. It prevents oxidative damage and arsenic-induced apoptosis by suppressing endoplasmic reticulum stress and enhancing mitochondrial function. MEL may represent a potential therapeutic option for arsenite-induced peripheral neuropathy.	[89]
		MEL at 1 mM, following a 2 h pre-incubation, protects cortical neurons exposed to various concentrations of NiCl_2_ for 12 h. MEL reduces reactive oxygen species production, improves mitochondrial function and cell viability, preserves mitochondrial membrane potential, and maintains mtDNA content in nickel-treated neurons.	[91]
		Preincubation with a 1 μM melatonin solution for 12 h effectively protected cells from cobalt (300 μM)-induced cytotoxicity and oxidative stress. Exposure of SH-SY5Y cells to cobalt significantly increased β-amyloid release; however, melatonin treatment attenuated these deleterious effects, highlighting its neuroprotective potential.	[92]
		MEL at a concentration of 1 mM provided significant neuroprotection against mercury-induced toxicity in SH-SY5Y neuroblastoma cells. A 12 h preincubation with this dose effectively attenuated oxidative stress, reduced β-amyloid release, and prevented tau hyperphosphorylation induced by 180 nM HgCl_2_.	[93]
Neurogenesis	Alzheimer’s disease	MEL demonstrates strong neuroprotective effects in preclinical studies, particularly in inhibiting β-amyloid aggregation and tau phosphorylation. In clinical studies, lower doses (3–6 mg) improve sleep quality and may slow symptom progression; however, achieving neuroprotective effects is thought to require significantly higher doses (50–100 mg/day), which necessitates further clinical research in this area.	[102]
		MEL, through the upregulation of Bcl-2 expression and its antioxidant effects, may counteract neurodegeneration in Alzheimer’s disease, making it a promising candidate for further clinical investigation. These effects were observed at doses of 10 mg/kg/day administered orally in rats for 30 days—doses that translate to high equivalents in humans, falling within the neuroprotective rather than merely sleep-regulating range.	[119]
	Parkinson’s disease	MEL improves sleep and quality of life in patients with Parkinson’s disease in clinical studies at doses of 3–5 mg/day. These effects include reduced daytime sleepiness, shortened sleep latency, and increased total sleep time.	[164]
		In animal models, neuroprotective effects of MEL have been observed through inhibition of ferroptosis and regulation of circadian rhythms. These actions require higher doses (e.g., 10 mg/kg), which may be relevant for future translational research.	[155]
	Huntington’s disease	MEL at a 10^−5^ M concentration prevents lipid peroxidation, total LDH activity, GSH depletion, and reduced antioxidant enzyme activity in N1E-115 cells incubated with 100 mM 3-nitropropionic acid.	[173]
		Melatonin at a dose of 10 mg/kg administered intraperitoneally 30 min and 60 min after 3-nitropropionic acid injection reduces lipid peroxidation and oxidative protein damage in rats.	[175]
		MEL administered intraperitoneally at a dose of 30 mg/kg delays disease onset and mortality in a transgenic mouse model of HD (and extends survival by 21%). Its interaction with the mitochondrial receptor MT1 is crucial for neuroprotection.	[67]

Abbreviations: AFMK—1N-acetyl-2N-formyl-5-methoxy-nuramine; AKT—protein kinase B; AMK—1N-acetyl-5-methoxy-nuramine; AMPK—AMP-activated protein kinase; BDNF—brain-derived neurotrophic factor; CAT—catalase; c3-OHM—cyclic 3-hydroxymelatonin; CREB—cAMP response element-binding protein; Drp1—dynamin-related protein 1; ERRα—estrogen-related receptor alpha; Fis1—mitochondrial fission 1 protein; GPx—glutathione peroxidase; GSH—glutathione; HD— Huntington’s disease; IL-33—interleukin 33; LDH—lactate dehydrogenase; MAPK—mitogen-activated protein kinase; mtDNA— mitochondrial DNA; MEL—melatonin; mPTP—mitochondrial permeability transition pore; MT2—melatonin receptor type 2; NMDA—N-methyl-D-aspartate receptor; Nrf2—nuclear factor erythroid 2-related factor 2; 6-OHM—6-hydroxymelatonin; PKA—protein kinase A; PI3K—phosphoinositide 3-kinase; PGC-1α—peroxisome proliferator-activated receptor-gamma coactivator 1-alpha; PLC—phospholipase C; ROS—reactive oxygen species; RNS—reactive nitrogen species; SOD—superoxide dismutase; SIRT1/3—Sirtuin 1/3 (silent mating type information regulation 2 homolog 1/3).

## Abbreviations

The following abbreviations are used in this manuscript:

AD	Alzheimer’s disease
HD	Huntington’s disease
PD	Parkinson’s disease
ETC	Electron transport chain
OXPHOS	Oxidative phosphorylation
mtDNA	Mitochondrial DNA
MEL	Melatonin (N-acetyl-5-methoxytryptamine)
SCN	Suprachiasmatic nucleus
GABA	Gamma-aminobutyric acid
SCG	Superior cervical ganglion
NO	Nitric oxide
mPT	Mitochondrial permeability transition
mPTP	Mitochondrial permeability transition pore
PLC	Phospholipase C
AC	Adenylate cyclase
AADC	L-aromatic amino acid decarboxylase
AA-NAT	Arylalkylamine N-acetyltransferase
ASMT	N-acetylserotonin-O-methyltransferase
TPH	Tryptophan-5-hydroxylase
ROS/RNS	Reactive oxygen and nitrogen species
AFMK	1N-acetyl-2N-formyl-5-methoxy-nuramine
AMK	1N-acetyl-5-methoxy-nuramine
c3-OHM	Cyclic 3-hydroxymelatonin
ABTS	2,2′-azino-bis(3-ethylbenzthiazoline-6-sulfonic acid)
6-OHM	6-hydroxymelatonin
O_2_^•−^	Superoxide anion
^•^OH	Hydroxyl radical
^1^O_2_	Singlet oxygen
H_2_O_2_	Hydrogen peroxide
HOCl	Hypochlorous acid
ROO^•^	Perixyl radical
ONOO^−^	Peroxynitrite anion
NO^+^	Nitrosonium ion
CO_3_^•−^	Carbonate radical
2-OHM	2-Hydroxymelatonin
4-OHM	4-Hydroxymelatonin
IDO	Indoleamine 2,3-dioxygenase
MPO	Myeloperoxidase
HRP	Hemoperoxidases
AMMC	3-acetamidomethyl-6-methoxycinnolinone
AMNK	1N-acetyl-5-methoxy-3-nitrokynuramine
MQA	N-[2-(6-methoxyquinazolin-4-yl)-ethyl]-acetamide
BBB	Blood–brain barrier
GPx	Glutathione peroxidase
CAT	Catalase
SOD	Superoxide dismutase
AKT	Protein kinase B
BDNF	Brain-derived neurotrophic factor
cAMP	Cyclic adenosine monophosphate
CaM	Calmodulin
CaMKII	Calcium/calmodulin-dependent protein kinase II
CREB	cAMP response element-binding protein
DAG	Diacylglycerol
ERK 1/2	Extracellular signal-regulated kinases
IKK	IκB kinase
IκB	Inhibitor of nuclear factor kappa B
IP3	Inositol trisphosphate
MAPK	Mitogen-activated protein kinase
MT1/MT2	Melatonin receptor type 1 and type 2
NF-κB	Nuclear factor-kappa B
AP-1	Activator protein-1
Nrf2	Nuclear factor erythroid 2-related factor 2
ARE	Antioxidant response element
PI3K	Phosphoinositide 3-kinase
PIP2	Phosphatidylinositol 4,5-bisphosphate
PIP3	Phosphatidylinositol 3,4,5-trisphosphate
PKA	Protein kinase A
PKC	Protein kinase C
SIRT1/3	Sirtuin 1/3 (Silent mating type information regulation 2 homolog 1/3)
Cyt C	Cytochrome C
UQ	Ubiquinone
PEPT1/2	Human proton-coupled oligopeptide transporters
Δψm	Mitochondrial membrane potential
AMPK	5′ Adenosine monophosphate-activated protein kinase
PGC-1α	Peroxisome proliferator-activated receptor-gamma coactivator 1-alpha
ERRα	Estrogen-related receptor alpha
Drp1	Dynamin-related protein 1
Fis1	Mitochondrial fission protein 1
OPA1	Optic atrophy 1
RCR	Respiratory control ratio
mHTT	Mutant huntingtin
AMD	Age-related macular degeneration
RPE	Retinal pigment epithelial
nNOS	Neuronal nitric oxide synthase
iNOS	Inducible nitric oxide synthase
PDH	Pyruvate dehydrogenase
PDK	Pyruvate dehydrogenase kinase
GR	Glutathione reductase
8-OHdG	8-hydroxy-2′-deoxyguanosine
HWR	Haber–Weiss reaction
Aβ	β-amyloid
APP	Amyloid precursor protein
ADAM10	Metalloproteinase domain-containing protein 10
BACE1	β-secretase β-site APP cleaving enzyme 1
PS1	Presenilin-1
REM	Rapid eye movement
BMAL1	Muscle ARNT-like 1
PER2	Period circadian protein homolog 2
NADPH	Nicotinamide adenine dinucleotide phosphate oxidase
IL-10	Interleukin-10
mTOR	Mechanistic target of rapamycin reduction
IRS-1	Insulin receptor substrate-1
IGF-1	Insulin-like growth factor 1
5-LOX	5-lipoxygenase-activating protein
LC3-II/p62	Microtubule-associated protein 1A/1B-light chain 3
FOXO	Forkhead box protein O
Wnt/β	Canonical Wnt/β-catenin signaling pathway
NMDAR	N-methyl-D-aspartate receptor
ChAT	Choline acetyltransferase
CHT	High-affinity choline transporter
VAChT	Vesicular acetylcholine transporter
M1R	Muscarinic M1 receptor
AChE	Acetylcholinesterase
NGF	Nerve growth factor
CNS	Central nervous system
HO-1	Heme oxygenase-1
TLR4	Toll-like receptor 4
MyD88	Myeloid differentiation primary response 88
COX-2	Cyclooxygenase-2
IL-1β	Interleukin-1β
IL-18	Interleukin-18
PSQI	Pittsburgh Sleep Quality Index
MSNs	Medium spiny neurons
CAG	Cytosine–adenine–guanine
Poly-Q	Polyglutamine tail
